# Dynamic expression of genes associated with schizophrenia and bipolar disorder across development

**DOI:** 10.1038/s41398-019-0405-x

**Published:** 2019-02-04

**Authors:** Nicholas E. Clifton, Eilís Hannon, Janet C. Harwood, Arianna Di Florio, Kerrie L. Thomas, Peter A. Holmans, James T. R. Walters, Michael C. O’Donovan, Michael J. Owen, Andrew J. Pocklington, Jeremy Hall

**Affiliations:** 1 Neuroscience and Mental Health Research Institute, Cardiff University, Cardiff, UK; 2 MRC Centre for Neuropsychiatric Genetics and Genomics, Division of Psychological Medicine and Clinical Neurosciences, Cardiff University, Cardiff, UK; 3 University of Exeter Medical School, University of Exeter, Exeter, UK

## Abstract

Common genetic variation contributes a substantial proportion of risk for both schizophrenia and bipolar disorder. Furthermore, there is evidence of significant, but not complete, overlap in genetic risk between the two disorders. It has been hypothesised that genetic variants conferring risk for these disorders do so by influencing brain development, leading to the later emergence of symptoms. The comparative profile of risk gene expression for schizophrenia and bipolar disorder across development over different brain regions however remains unclear. Using genotypes derived from genome-wide associations studies of the largest available cohorts of patients and control subjects, we investigated whether genes enriched for schizophrenia and bipolar disorder association show a bias for expression across any of 13 developmental stages in prefrontal cortical and subcortical brain regions. We show that genetic association with schizophrenia is positively correlated with expression in the prefrontal cortex during early midfetal development and early infancy, and negatively correlated with expression during late childhood, which stabilises in adolescence. In contrast, risk-associated genes for bipolar disorder did not exhibit a bias towards expression at any prenatal stage, although the pattern of postnatal expression was similar to that of schizophrenia. These results highlight the dynamic expression of genes harbouring risk for schizophrenia and bipolar disorder across prefrontal cortex development and support the hypothesis that prenatal neurodevelopmental events are more strongly associated with schizophrenia than bipolar disorder.

## Introduction

Schizophrenia and bipolar disorder both have their peak onset in early adulthood. It has been argued for some time that schizophrenia arises as a result of pathological changes occurring during early neurodevelopment^1–3^. One influential hypothesis is that at least some of the aetiological events occur during prenatal brain development, but their effects remain latent and are only expressed later as symptoms in the context of brain maturation^1,4^. Prefrontal cortical regions, which do not fully mature until the late second decade of life, have been proposed to be the locus of early developmental vulnerability for later schizophrenia^1,4,5^. The importance of prenatal neurodevelopment in risk for schizophrenia is supported by epidemiological evidence showing that prenatal and perinatal events such as infection, famine and obstetric complications can increase risk for the disorder, and by the observation that children with subtle neurological, cognitive and behavioural impairments are at enhanced risk for later developing the condition^3,6–8^.

There is less evidence that early neurodevelopmental events play an important role in the development of bipolar disorder^7,9^. In general, fewer epidemiological studies have examined associations between prenatal/perinatal events such as maternal infection and obstetric complications and risk for later bipolar disorder^9^. While some evidence exists for a role of prenatal influenza infection and low birth weight or prematurity in risk for bipolar disorder, the results have not been consistent across studies^9–15^. Furthermore, there is not strong evidence that individuals who later develop bipolar disorder had poorer premorbid cognitive functioning and social adjustment or increased rates of subtle neurological symptoms^7,16^.

There has also been interest in the potential role of developmental events occurring postnatally during childhood and adolescence in the development of adult onset psychiatric conditions such as schizophrenia and bipolar disorder^5,7,17^. It is well established that extensive neuronal maturation occurs across childhood and adolescence^5^. In particular, prefrontal cortical areas show considerable developmental change across late childhood and adolescence with extensive synaptic pruning and elimination of excitatory synapses shaping the late-maturing cortex^5,18^. There is also evidence that environmental risk factors operating across the period of childhood and adolescence can increase risk for both schizophrenia and bipolar disorder, for example, severe childhood abuse has been associated with an increased risk of both disorders^19–21^, and maternal loss prior to the age of 5 has been associated with bipolar disorder^22^, while cannabis use has been reported to have greater effects on risk for the development of psychosis if exposure occurs prior to or during the adolescent period^5,23,24^. One difficulty in interpreting the salience of these findings is that the causal nature of these candidate risk factors has yet to be firmly established.

Both schizophrenia and bipolar disorder have a high degree of heritability^25,26^. Recent large-scale genomic studies have made major progress in determining the genetic architecture of schizophrenia and bipolar disorder^27–29^. Genetic risk for both conditions is highly polygenic, the risk architectures including a large number of common variants, which collectively account for a significant proportion of heritability for these conditions^30,31^. There is considerable, although not complete, overlap seen between common variants conferring risk for schizophrenia and bipolar disorder as identified in genome-wide association studies^32,33^. However, rarer but more penetrant genomic risk variants, such as copy number variants, are generally found to be more enriched in schizophrenia than in bipolar disorder^34,35^. As the rare mutations that contribute to schizophrenia also contribute to neurodevelopmental disorders including intellectual disability, autism, ADHD and developmental delay, these findings have contributed to the development of a “neurodevelopmental continuum” model in which schizophrenia is considered to be associated with a greater load of early neurodevelopmental insults than bipolar disorder^16,34,35^.

Previous studies examining the expression of the top genome-wide association study (GWAS) common variant risk-associated loci for schizophrenia in post-mortem tissue have supported a high level of expression in prenatal brain, consistent with the view that risk for schizophrenia may have early neurodevelopmental origins^4,36–39^. However, less is known about the early expression of bipolar-associated genes, or the comparative profile of risk gene expression for schizophrenia and bipolar disorder across development and into adulthood. Here, we have used data from large-scale GWAS of schizophrenia and bipolar disorder combined with gene expression data across development from the BrainSpan data set^40,41^, to examine the dynamic expression of genes harbouring common risk variants across developmental stages from prenatal life to adulthood.

## Methods

### Neurodevelopmental transcriptome

Transcriptomic data were obtained from the Allen Institute BrainSpan Atlas:^40^ a resource providing RNA sequencing-derived gene expression data of post-mortem brain tissue from 38 individuals between 8 weeks post-conception and 40 years of age (Supplementary Table 1). The data were filtered to remove samples with low RNA integrity (RIN < 7). Genes that were unexpressed (zero reads per kilobase per million (RPKM) in all samples) or did not possess an NCBI/Entrez gene ID were excluded, as were duplicate entries. RPKM gene expression values were adjusted to control for RNA integrity, ethnicity and gender by fitting a linear regression model of expression with these factors as independent variables; the residuals and intercepts from this model were used as the adjusted expression values. Subjects were grouped into 13 developmental stages (Supplementary Table 1).

### Calculation of gene expression scores

For each developmental stage, each gene was assigned a relative expression score reflecting the degree of expression relative to all other developmental stages. Expression scores were calculated by fitting a linear regression model for each gene and developmental stage: expression = brain region + developmental stage, where expression is the adjusted expression value of the gene and developmental stage is a binary variable denoting whether the sample was from the stage or not. This model was fitted to selected groups of brain regions. The brain region covariate was included to account for differences in gene expression between subregions. Using this model, we defined the expression score = *R*^2^ × sgn(coefficient), so that for a given developmental stage: genes with high expression relative to other stages will have large positive scores; those with low expression relative to other stages will have large negative scores; and those whose expression is relatively constant across development will have scores close to zero. We structured analyses in a hierarchical fashion whereby developmental expression was first analysed using the combined data from all brain regions; a second tier of analyses was then performed on major groups of subregions.

### Genotype data

Schizophrenia and bipolar disorder single-nucleotide polymorphism (SNP) association summary statistics were taken from previously described GWAS meta-analyses^27,28^. A combined schizophrenia sample of 40,675 cases and 64,643 control subjects consisted of 11,260 cases and 24,542 controls from studies of UK patients with schizophrenia taking clozapine (CLOZUK)^27^, and 29,415 cases and 40,101 controls from a large-scale GWAS performed by the Psychiatric Genomics Consortium (PGC)^29^. The bipolar disorder sample comprised of 20,352 cases and 31,358 controls from 32 cohorts of European descent, compiled as part of a recent PGC GWAS^28^.

Schizophrenia and bipolar disorder GWAS SNPs were filtered to include only those with a minor allele frequency ≥0.01 and imputed INFO score ≥ 0.6. Additional filters were applied to exclude the extended major histocompatibility complex (xMHC) region and the X chromosome, consistent with previous analyses^27^.

### Gene property analysis

The relationship between gene expression scores and enrichment for association with schizophrenia or bipolar disorder was determined for each developmental stage using gene property analyses in MAGMA v1.06^42^. Briefly, common SNP association *P*-values were combined into gene-wide *P*-values (via the MAGMA SNP-wise mean model), using a window of 35 kb upstream and 10 kb downstream^43^ of each gene in order to include SNPs within regulatory regions. Only protein-coding genes were included in the analysis. The gene property analysis method performs a linear regression of gene-wide association against a gene-level property (here, relative expression score), in which covariates are included to correct for potential confounds. Our analyses were two-tailed and included correction for gene size and SNP density. In conditional analyses, gene expression scores for the conditioned developmental stage were included as covariates. At each tier of analysis, *P*-values were Bonferroni adjusted for the number of developmental stages or brain regions analysed.

### Pathway analysis

To identify functional processes enriched among genes with the most extreme relative expression, functional gene annotations were compiled separately from the Gene Ontology (GO)^44^ and Mouse Genome Informatics (MGI) Mammalian Phenotype (MP)^45^ databases (4th July 2018). GO annotations derived from the following evidence codes were excluded: IEA (Inferred from Electronic Annotation); NAS (Non-traceable Author Statement); and RCA (inferred from Reviewed Computational Analysis).

To identify sets of genes with which to perform pathway analyses, all expressed genes were ranked by their expression during the developmental stage of interest then divided into ten equal bins. Each decile was tested for genetic association enrichment via a two-tailed competitive test in MAGMA^42^. Significantly associated deciles (following correction for ten tests) were then merged and tested for GO/MP term enrichment (GO and MP terms tested separately) relative to a background set of all expressed genes, using a Fisher’s exact test. Following Bonferroni correction for multiple testing of all GO or MP terms, significantly enriched terms were subjected to a refinement procedure: deciles were re-tested for enrichment of each term following the removal of genes from another term. This was done in a step-wise fashion whereby genes from the smallest term were removed first and any terms of weaker enrichment that were no longer significant on re-test (determined by uncorrected *P*-value in Fisher’s exact test) were removed from the analysis.

To investigate whether genes corresponding to refined GO/MP terms were more enriched for genetic association than other genes in significantly associated deciles, genes common to both refined terms and significant decile(s) were subjected to one-tailed gene-set association analyses conditioning on all genes within the significant decile(s).

## Results

### Whole-brain developmental expression profile of genes enriched for association with schizophrenia and bipolar disorder

**Fig. 1:**
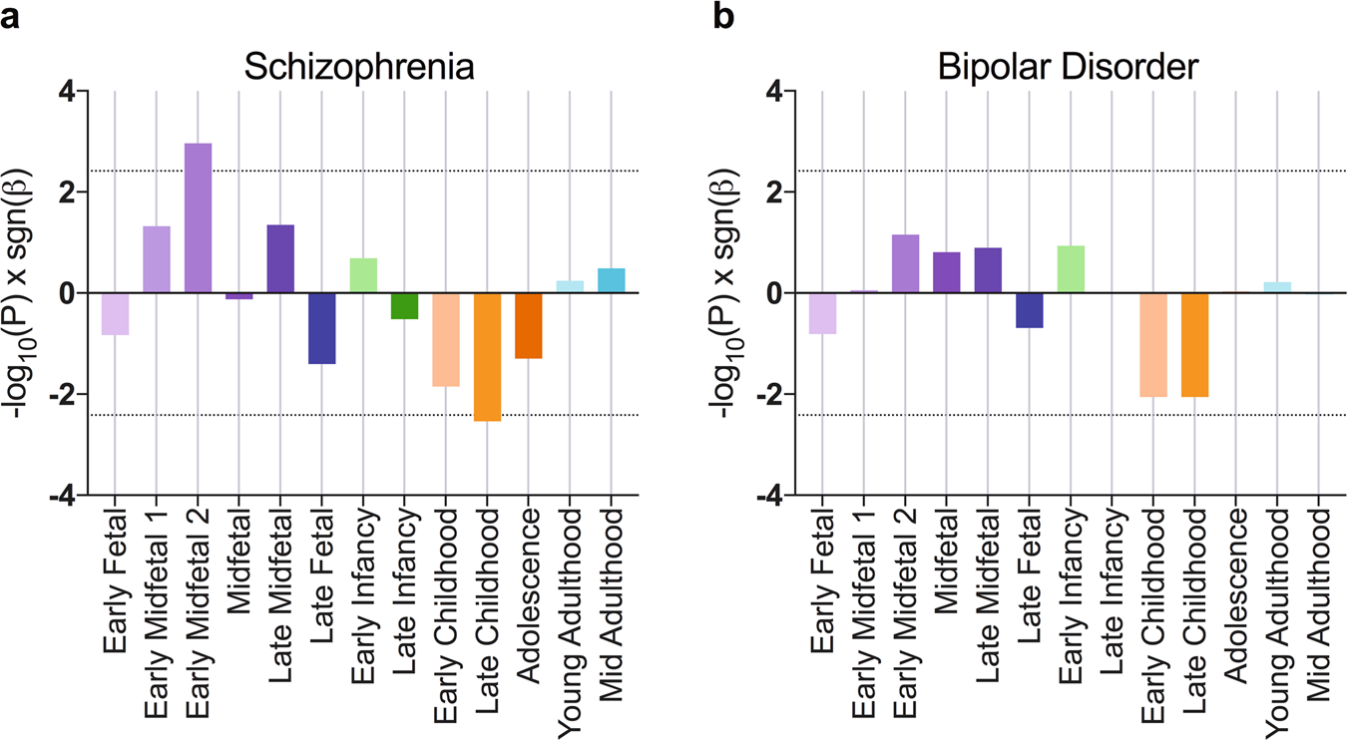
Brain-wide relationships between expression and gene-level evidence of association with schizophrenia and bipolar disorder. MAGMA gene property analysis of gene expression scores derived from RNA-sequencing data across all brain regions was performed against case-control common variant data for schizophrenia (**a**) and bipolar disorder (**b**). Bars represent −log_10_(*P*) sgn(*β*), where *P* is uncorrected following gene property analysis and *β* is the corresponding regression coefficient. Dotted lines show significance thresholds accounting for multiple testing across 13 developmental stages

We used gene property analysis in MAGMA^42^ to determine whether gene expression during particular developmental periods is correlated with enrichment for common variant association for schizophrenia or bipolar disorder. In a combined analysis of gene expression data from all brain regions, we observed a non-uniform relationship between relative gene expression and enrichment for schizophrenia association across development (Fig. 1a). Genetic association with schizophrenia was most strongly related to expression during the Early Midfetal 2 developmental stage (adjusted *P* = 0.014). Conversely, an inverse relationship between gene expression and schizophrenia association was observed at Late Childhood (adjusted *P* = 0.038), indicating that schizophrenia associated genes were relatively under-expressed at this time point; relative expression at other stages was non-significant.

Brain-wide analyses of bipolar disorder GWAS data showed some variation of risk-associated gene expression bias, although no significant relationship between gene expression and gene-wide association for bipolar disorder risk was observed at any single developmental stage (Fig. 1b).

### Cortical and subcortical expression of risk-associated genes across development

Since trajectories of neurodevelopmental gene expression vary considerably between brain regions^40^, we sought to determine whether relationships with genetic association for schizophrenia or bipolar disorder were more pronounced for specific subregions of the brain. We tested these relationships in grouped sets of prefrontal cortex (medial prefrontal cortex, dorsolateral prefrontal cortex, ventrolateral prefrontal cortex and orbital frontal cortex), non-prefrontal cortex (posterior superior temporal cortex, inferolateral temporal cortex, primary auditory cortex, primary visual cortex, primary motor cortex, posteroventral parietal cortex and primary somatosensory cortex) and subcortical regions (amygdaloid complex, thalamus, striatum and hippocampus).

**Fig. 2:**
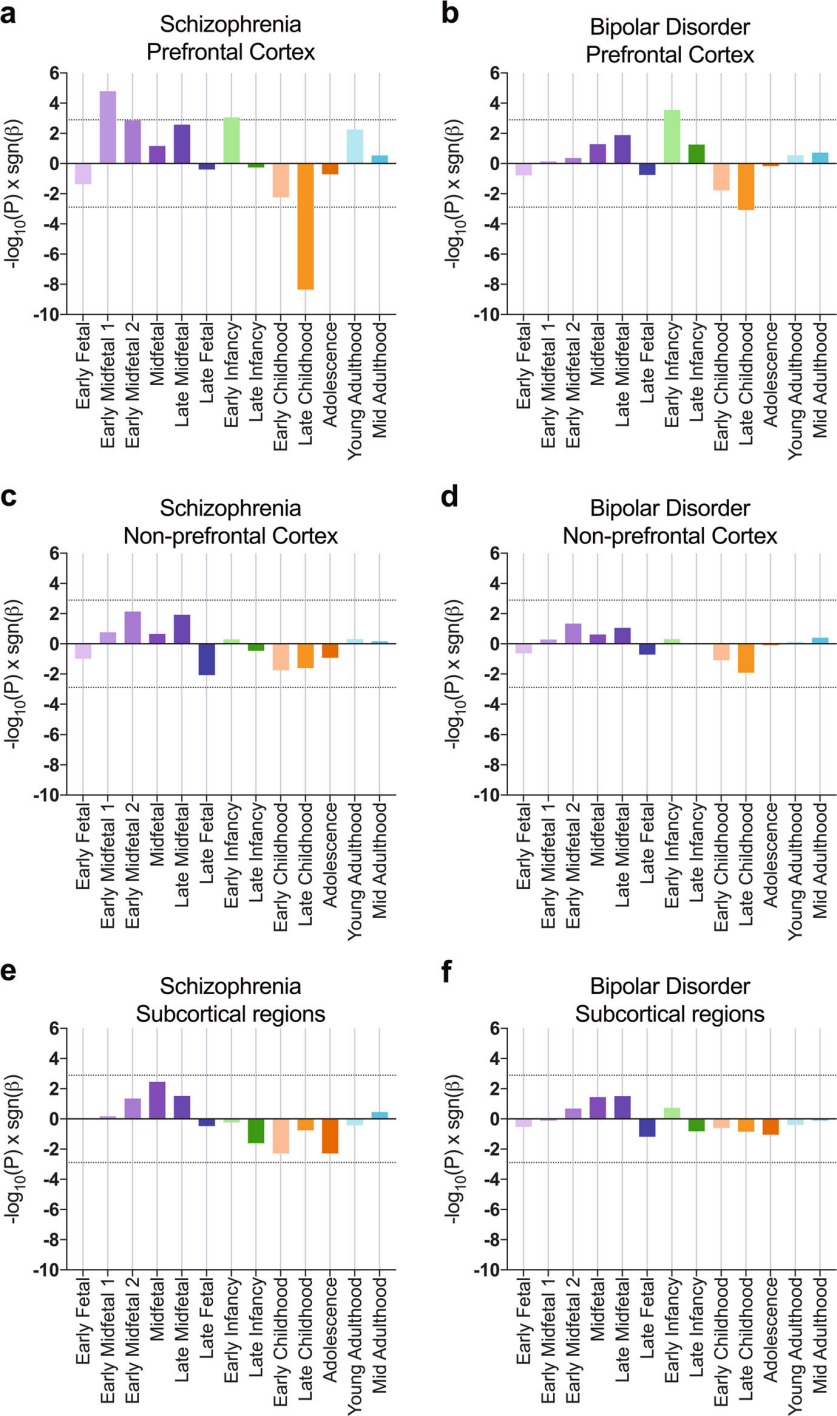
Cortical and subcortical relationships between gene expression and association with schizophrenia and bipolar disorder. Relationships between gene expression and psychiatric genetic association were determined from separate analyses of prefrontal cortex (**a**, **b**), non-prefrontal cortex (**c**, **d**) and subcortical regions (**e**, **f**). Prefrontal Cortex includes medial prefrontal cortex, dorsolateral prefrontal cortex, ventrolateral prefrontal cortex and orbital frontal cortex. Non-prefrontal cortex includes posterior superior temporal cortex, inferolateral temporal cortex, primary auditory cortex, primary visual cortex, primary motor cortex, posteroventral parietal cortex and primary somatosensory cortex. Subcortical regions include amygdaloid complex, thalamus, striatum and hippocampus. Bars represent −log_10_(*P*) sgn(*β*), where *P* is uncorrected following gene property analysis and *β* is the corresponding regression coefficient. Dotted lines show significance thresholds accounting for multiple testing for 13 developmental stages and three subregion groups

For schizophrenia, we observed increased expression of more highly associated genes in prefrontal cortex at Early Midfetal 1 (adjusted *P* = 6.3 × 10^–4^) and Early Infancy (adjusted *P* = 0.034; Fig. 2a). Gene expression at Early Midfetal 2 development was nominally related to schizophrenia association (adjusted *P* = 0.053). Consistent with whole-brain analyses, gene expression in prefrontal cortex at Late Childhood was strongly negatively correlated with schizophrenia association (adjusted *P* = 1.7 × 10^–7^). In non-prefrontal cortex and subcortical regions, gene expression was not correlated with schizophrenia association at any developmental stage (Fig. 2c, e).

Subregion analyses revealed that genetic association with bipolar disorder was modestly correlated with higher prefrontal cortical expression during Early Infancy (adjusted *P* = 0.011), and lower prefrontal cortex expression during Late Childhood (adjusted *P* = 0.033; Fig. 2b). While these postnatal expression profiles are similar to those observed for genetic association with schizophrenia, prenatal expression did not correlate with association for bipolar disorder. Gene expression in non-prefrontal cortex or subcortical regions did not correlate with bipolar disorder association at any developmental stage (Fig. 2d, f).

### Comparison of genetic association between developmental stages

We next investigated whether genes mediating the relationship between common variant association and prefrontal cortical expression during early developmental stages were also responsible for the relationship found in Late Childhood. To determine whether these signals are driven by overlapping sets of genes, we performed conditional gene property analyses for the relevant developmental stages.

For schizophrenia, conditioning on (low) Late Childhood expression resulted in reduced correlation of prefrontal cortical expression at Early Midfetal 1 with common variant association (uncorrected *P* = 0.0054). This indicates that a substantial proportion of risk-associated genes, which have high prefrontal cortex expression during this prenatal developmental stage tend to have subsequent low prefrontal expression in this region during Late Childhood. In the reciprocal analysis, conditioning on Early Midfetal 1 had little effect on the strong negative correlation between Late Childhood gene expression and schizophrenia association (uncorrected *P* = 1.2 × 10^–6^), implying that there is a larger set of independently associated genes with particularly low expression during this stage or a sizeable set of highly expressed genes depleted for association.

For both schizophrenia and bipolar disorder, conditioning on Late Childhood did not ablate the relationship between gene expression during Early Infancy and genetic association (schizophrenia uncorrected *P* = 0.0024; bipolar disorder uncorrected *P* = 5.9 × 10^–4^), suggesting that during Early Infancy an independent set of associated genes are differentially expressed.

### Functional analysis of expressed genes

**Fig. 3:**
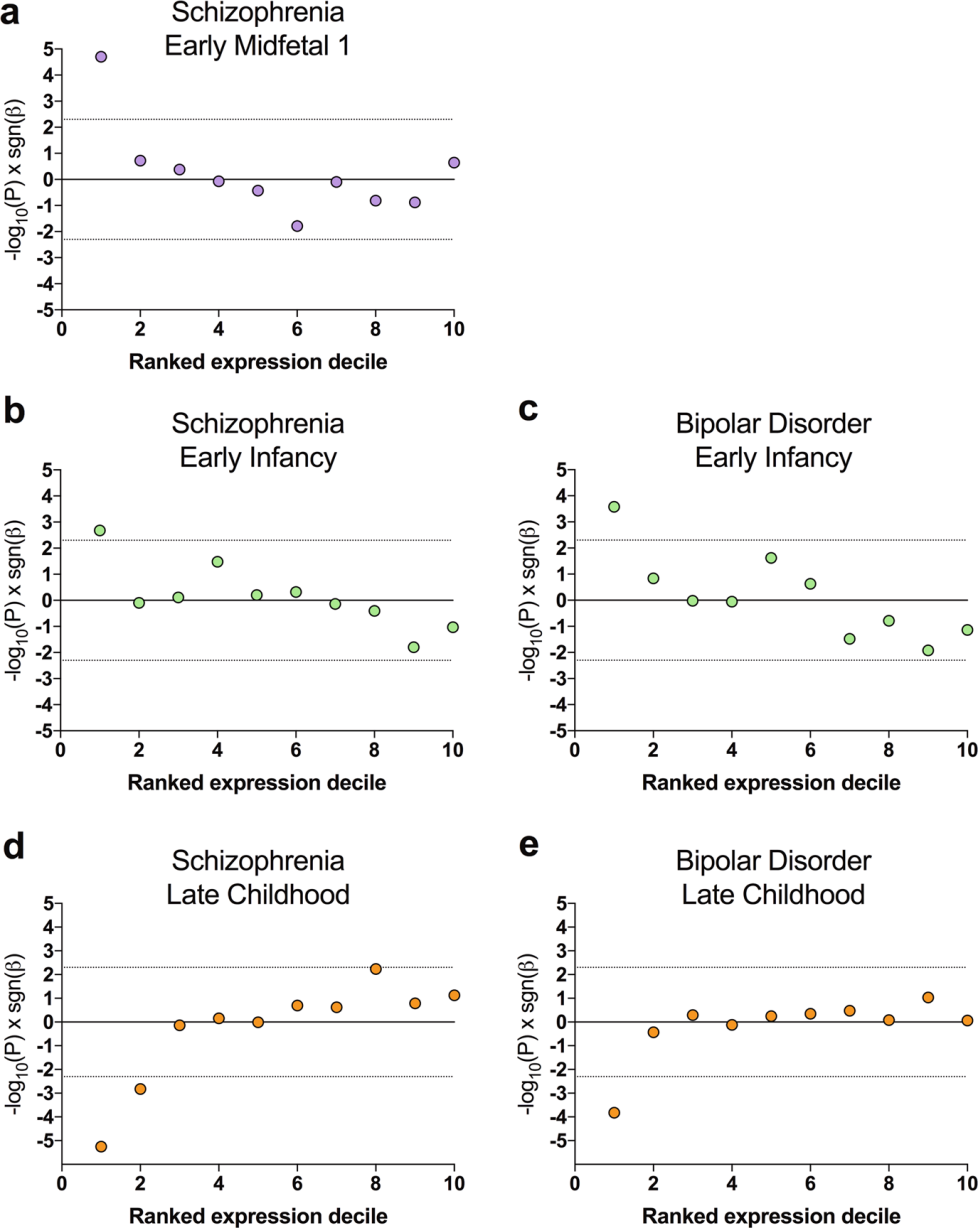
Distribution of enrichment for association with schizophrenia or bipolar disorder across expression deciles. Gene sets ranked by prefrontal cortical expression during Early Midfetal 1 (**a**), Early Infancy (**b**, **c**) and Late Childhood (**d**, **e**) were tested for enrichment for association with schizophrenia (**a**, **b** **d**) or bipolar disorder (**c**, **e**). All expressed genes were divided into ten equal groups ranked by their expression. Points represent −log_10_(*P*) sgn(*β*), where *P* is uncorrected following gene-set enrichment analysis and *β* is the corresponding regression coefficient. Dotted lines delimit significance thresholds following correction for ten analyses

To investigate whether the associations identified between developmental expression and genetic risk implicate specific functional processes in disease, we first identified sets of genes driving the relationship between prefrontal cortical expression and common variant association with schizophrenia and bipolar disorder. Testing deciles of all genes ranked by expression, we found that only the top 10% or 20% of genes expressed at each developmental stage highlighted by the previous analyses (Early Midfetal 1, Early Infancy or Late Childhood) were significantly enriched (or in the case of Late Childhood, significantly depleted) for common variant association (Fig. 3).

Functional gene-set enrichment analysis was then used to identify functional categories of genes enriched in these significantly associated deciles (Supplementary Tables 2–5). For Early Midfetal 1 and Early Infancy, these enriched GO/MP terms were then tested for evidence of higher genetic association than other genes in significant deciles. Since significant expression deciles for Late Childhood were depleted for association with schizophrenia and bipolar disorder, we probed enriched functional terms for evidence of *lower* association than other genes in these deciles, to potentially identify functional processes occurring at this stage of development that *do not* contribute to psychiatric risk. No individual functional term was significantly enriched or depleted for association following correction for multiple testing (Supplementary Tables 5–10). Either the specific pathophysiological processes are not well captured by the functional annotations we have analysed, or genetic risk factors may be diffused across a wide range of biological processes affecting prefrontal cortical development at these time-points.

## Discussion

In this study, we have examined the relationship between gene expression and enrichment for association with schizophrenia and bipolar disorder across development using post-mortem gene expression data from the BrainSpan database^40,42^. Our results show that gene-level association with schizophrenia correlated with higher relative expression in early midfetal development and early infancy, and negatively correlated with expression during late childhood. In contrast, in bipolar disorder we found no evidence of increased expression of associated genes in the prenatal period, although we observed a similar pattern of change in the expression of genes associated with bipolar disorder in prefrontal cortex across infancy and childhood to that seen in analyses of schizophrenia association.

Our finding of increased expression of genes associated with schizophrenia in the prenatal period is consistent with previous reports^4,36–38^. In the present study, we have examined this enrichment across the full polygenic signal of schizophrenia association using gene property analyses in MAGMA, suggesting that this bias towards prenatal expression exists across a wide number of schizophrenia associated alleles. Our results show high relative expression of schizophrenia- associated genes in the prefrontal cortex during early midfetal development, consistent with findings from key epidemiological studies implicating this period in risk for schizophrenia^3,6–8^. This period of development is one of profound neurogenesis and corticogenesis^5^. Subtle alterations in gene expression modulating these processes could represent one form of the early developmental cortical “lesion” proposed by the neurodevelopmental hypothesis of schizophrenia^1,4^. Notably a similar prenatal bias in expression of risk-associated genes has previously been described for autism and intellectual disability^46^, further supporting the view that these disorders can collectively be considered to be neurodevelopmental in origin. However, for genes associated with bipolar disorder the elevation in fetal expression was less pronounced and we failed to observe any significant evidence of a prenatal bias in gene expression, consistent with the view that these conditions can be collectively viewed as lying on a spectrum of neurodevelopmental risk, with bipolar disorder characterised by a lower prenatal burden of risk^7,34,35^.

The transition from late childhood into adolescence also represents a profound period of frontal cortical maturation. In particular, this period is characterised by the elimination (“pruning”) of excitatory synapses in the frontal cortex resulting in changes in excitatory-inhibitory balance and dopaminergic regulation and the emergence of adult patterns of executive functioning^5,24^. It has for some time been hypothesised that risk for schizophrenia and related disorders may be in part mediated through impacts on these late maturational changes in the frontal cortex, which immediately precede the period of peak onset of psychotic disorders^17^. In the present study, we find evidence that in both schizophrenia and bipolar disorder there is a profound change in the expression of associated genes in frontal cortex across the transition from late childhood into adolescence. The pattern in both disorders is of relatively low expression in late childhood, which then normalises at the transition into adolescence. While we do not see an overall differential expression of associated genes in adolescence, these data do suggest that there is a shift in expression for both schizophrenia- and bipolar-associated genes across this developmental period. It is also notable that a similar pattern of gene expression change is seen in both schizophrenia and bipolar disorder. This finding is consistent with the strong genetic overlap of these conditions and suggests that this overlap derives in part from genes, which show dynamic expression changes in the frontal cortex across later childhood and adolescence.

We also found evidence of an increase in expression of genes enriched for association with both bipolar disorder and schizophrenia in frontal cortex during early infancy. Early infancy is a critical period of human brain development, but has been less discussed in terms of later risk for psychiatric disorders such as bipolar disorder and schizophrenia than prenatal and adolescent development. Human infancy represents a period of rapid brain maturation including synaptogenesis, myelination and the establishment of patterns of network activation, which include frontal regions^47^. These developmental changes sub-serve the emergence of cognitive functions including early language development, attention, working memory and self-regulation^48^, all of which are strongly implicated in neuropsychiatric disorders. Interestingly, our conditional analyses show that the genes driving this increase in expression during infancy are independent to those driving the negative correlation between expression and psychiatric disorder in later childhood. This contrasts with our analysis investigating potential overlaps between genes expressed in prenatal development in schizophrenia and those showing changes across late childhood-adolescence where we find evidence of a substantial overlap in implicated genes through conditional analyses.

Our study has a number of limitations. Firstly, differences in power attributed by patient sample sizes may contribute to apparent differences between schizophrenia and bipolar disorder. Furthermore, the developmental expression data come from a relatively small group of post-mortem brains, although this data set is large compared to many comparator datasets and benefits from high quality standards and careful curation^40,49^. Nevertheless, replication of the current findings in additional samples will be important. Such further analyses may also facilitate a higher resolution of subregion analysis; the present study was unable to pinpoint developmental correlations of risk- associated genes to subregions of the prefrontal cortex (data not shown), which may be due to either the generalisation of these findings to the whole region, or a lack of sufficient sample size. In addition, it will be desirable to identify which specific cell types the enrichment signals derive from, given increasing evidence of the involvement of specific key cell types in schizophrenia and related disorders^50,51^. While our study highlights functional groups of genes co-expressed during critical developmental periods, we were unable to pinpoint discrete molecular pathways contributing to our principal findings. Further work is required to substantiate whether genetic risk is concentrated within specific pathways contributing to brain development at these time-points. Finally, the use of post-mortem gene expression data, while powerful, does not capture activity-dependent changes in gene expression, which may be particularly important in the context of adult plasticity and learning.

Overall, this work highlights the dynamic nature of expression of association-enriched genes for schizophrenia and bipolar disorder across development. Our results are consistent with the view that there is a significant early neurodevelopmental component to risk for schizophrenia, deriving from processes operating in prenatal development, which does not appear to be extensively shared with bipolar disorder. We also provide evidence of a common pattern of changes in expression of genes enriched for association with both schizophrenia and bipolar disorder across infancy, childhood and adolescence. Our results show a particularly strong association between the expression of associated genes in both conditions and prefrontal cortical development, supporting the view that the dynamic and extended development of the human prefrontal cortex is an important substrate for risk for these disorders. Overall our data are consistent with the view that schizophrenia and bipolar disorder lie on a continuum of neurodevelopmental risk^34,35^, with evidence of substantial similarities in risk gene expression across development but a greater burden of schizophrenia risk within genes expressed during prenatal development.

## Supplementary information

Supplementary Information

Supplementary Tables 2-10
