# Supplementary material for: Dynamic expression of genes associated with schizophrenia and bipolar disorder across development
Source: Transl Psychiatry. 2019 Feb 4;9:74. doi: 10.1038/s41398-019-0405-x (PMC6362023; doi:10.1038/s41398-019-0405-x)
Supplement: Supplementary file 1 — Supplementary Information [file 41398_2019_405_MOESM1_ESM.pdf]

Developmental Stage	Ages	Age N	Stage N
**Early Fetal**	8 pcw	1	2
	9 pcw	1	
**Early Midfetal 1**	12 pcw	3	3
**Early Midfetal 2**	13 pcw	3	3
**Midfetal**	16 pcw	3	4
	17 pcw	1	
**Late Midfetal**	19 pcw	1	3
	21 pcw	2	
**Late Fetal**	24 pcw	1	2
	26 pcw	1	
**Early Infancy**	4 mos	2	2
**Late Infancy**	10 mos	1	2
	12 mos	1	
**Early Childhood**	2 yrs	1	4
	3 yrs	2	
	4 yrs	1	
**Late Childhood**	8 yrs	2	3
	11 yrs	1	
**Adolescence**	13 yrs	1	4
	15 yrs	1	
	18 yrs	1	
	19 yrs	1	
**Young Adulthood**	21 yrs	1	2
	23 yrs	1	
**Mid Adulthood**	30 yrs	1	4
	36 yrs	1	
	37 yrs	1	
	40 yrs	1	

Supplementary Table 1 Post-mortem brain samples of the Allen Institute BrainSpan Atlas. Samples were organised into 13 groups based on developmental stage. Shown are the number of individuals per age (Age N) and per developmental stage (Stage N) from which RNA sequencing data was used, after filtering. Post-conceptual weeks (pcw); months (mos); years (yrs).

Supplementary Tables 2-5 Pathway analysis of genes highly expressed during Early Midfetal 1, Early Infancy or Late Childhood. Gene expression deciles significantly associated with schizophrenia or bipolar disorder were tested for enrichment of genes annotated by Gene Ontology (GO) or Mammalian Phenotype (MP). *P*-values were adjusted (Padj) for multiple testing using Bonferroni correction. Highlighted terms remain after step-wise refinement for independently enriched gene sets, and were selected for genetic association analysis.

Supplementary Tables 6-10 Contributions of functionally-related genes to the relationship between prefrontal cortical expression during three developmental stages and common variant association with schizophrenia or bipolar disorder. Listed are gene sets annotated by GO or MP highly expressed during Early Midfetal 1, Early Infancy or Late Childhood. Gene set enrichment analyses were performed on genes common to both the term and the significant expression decile(s), with and without conditioning on the full significant expression decile(s). *P*-values and beta-values reflect the significance and effect size, respectively, of enrichment for common variant association with schizophrenia or bipolar disorder.
